# Family and Community Support, Brain-Derived Neurotrophic Factor, and Cognitive Performance in Older Adults: Findings From the Health, Wellbeing and Aging Study Population-Based Cohort

**DOI:** 10.3389/fnbeh.2021.717847

**Published:** 2021-09-21

**Authors:** Juliana Nery Souza-Talarico, Elke Bromberg, Jair Licio Ferreira Santos, Betania Souza Freitas, Diego Ferreira Silva, Yeda Aparecida Oliveira Duarte

**Affiliations:** ^1^College of Nursing, The University of Iowa, Iowa City, IA, United States; ^2^School of Nursing, University of Sao Paulo, São Paulo, Brazil; ^3^Department of Morphophysiological Sciences, Pontifical Catholic University of Rio Grande do Sul, Porto Alegre, Brazil; ^4^National Institute of Science and Technology for Translational Medicine (INCT-TM)/Brazilian National Research Council (CNPq), Ribeirão Preto, Brazil; ^5^Faculty of Medicine at Ribeirão Preto, University of Sao Paulo, São Paulo, Brazil; ^6^Faculty of Public Health, University of Sao Paulo, São Paulo, Brazil

**Keywords:** social support, family support, cognition, BDNF, aging

## Abstract

**Background:** Social networks can modulate physiological responses, protects against the detrimental consequences of prolonged stress, and enhance health outcomes. Family ties represent an essential source of social networks among older adults. However, the impact of family support on cognitive performance and the biological factors influencing that relationship is still unclear. We aimed to determine the relationship between family support, cognitive performance and BDNF levels.

**Methods:** Cross-sectional data from three-hundred, eight-six individuals aged on average 60 years enrolled in the Health, Wellbeing and Aging Study (SABE), a population-cohort study, were assessed for family support, community support and cognitive performance. Structural and functional family support was evaluated based on family size and interactions allied to scores in the Family APGAR questionnaire. Community assistance (received or provided) assessed the community support. Cognitive performance was determined using the Mini-Mental State Examination (MMSE), verbal fluency (animals per minute) and backward digital span. Blood samples were obtained to determine BDNF levels.

**Results:** Multivariate analysis showed that functional family support, but not structural, was associated with higher MMSE, verbal fluency and digit span scores, even controlling for potential cofounders (*p* < 0.001). Providing support to the community, rather than receiving support from others, was associated with better cognitive performance (*p* < 0.001). BDNF concentration was not associated with community support, family function, or cognitive performance.

**Conclusion:** These findings suggest that emotional components of functional family and community support (e.g., loving and empathic relationship) may be more significant to cognitive health than size and frequency of social interactions.

## Introduction

Prolonged exposure to stress mediators allied to maladaptive coping strategies is believed to influence the increased variability of cognitive functioning throughout adulthood and aging (Juster et al., 2010). Identifying protective factors capable of counterbalancing the negative impact of stress on the brain is critical to reducing the dementia burden worldwide.

The emerging literature on social support has shown that social networks and relationships modulate physiological responses (Uchino et al., 1996; Bowen and McGregor, 2014), protects against the detrimental consequences of stress (Heinrichs et al., 2003; McQuaid et al., 2016; Costa-Cordella et al., 2021), and enhance mental and physical health outcomes (Havranek et al., 2015; Ginting et al., 2016), through the use of active coping mechanisms when dealing with stressful life situations (Ozbay et al., 2007).

Social support is defined as structural and functional assistance/help that an individual can receive from family, friends, neighbors, and community members in times of need (Costa-Cordella et al., 2021). According to theoretical models of social support, structural assistance refers to the size and frequency of social interactions; while functional is the quality of relationships, which includes emotional (receiving love, encouragement and positive feedback) and instrumental components (provision of financial help, assistance with childcare, caregiving) (Southwick et al., 2005).

Longitudinal studies on the effect of perceived social support on health outcomes show that having significant companionship reduces the risk of heart disease and cardiovascular incidents (Anthony and O’Brien, 1999; Havranek et al., 2015; Ginting et al., 2016), respiratory diseases (Cohen et al., 2015) and strokes (Valtorta et al., 2016). In contrast, low social support has been associated with heightened stress reactivity, exaggerated autonomic and neuroendocrine responses to psychological stressors (Uchino et al., 1996).

In the brain, positive effects of social support on cognitive performance were described in older adults (Kelly et al., 2017; Costa-Cordella et al., 2021). Higher levels of social support have been associated with better cognitive functioning and less cognitive decline (Seeman et al., 2001; Kelly et al., 2017). Global cognition, executive functioning, working memory, episodic memory, attention and processing speed were associated with different components of social support such as social activity, social networks and social relationships (Kelly et al., 2017).

The biopsychological mechanisms underlying the positive relationship between social support and cognitive performance remain unclear. Specifically, qualitative and biological elements that underpin the relationship between social network and cognition is quite limited. For example, family ties represent an essential network source among older adults (Ying et al., 2020). A few studies investigated the impact of family support on cognitive performance with varied results (Windsor et al., 2014; Ge et al., 2017; Ying et al., 2020). Windsor et al. (2014) showed that positive exchanges with family were associated with less decline in perceptual speed, with that association attenuated by adjustment for physical functioning and depressive symptoms (Windsor et al., 2014). Ge et al. (2017) showed that higher strain from spouse and friends allied to higher support from friends, had significant associations with higher executive function (Ge et al., 2017). Evidence about the neurobiology basis explaining those associations is also scarce. Salinas et al. (2017) observed that having someone available to provide emotional support most or all of the time was associated with higher brain-derived neurotrophic factor (BDNF) and lower risk to develop dementia (Salinas et al., 2017). Brain-derived neurotrophic factor is a neuroprotective molecule critical for synaptic plasticity and neuronal repair (Emanueli et al., 2003), which is inducible by lifestyle factors and social enrichment (Neeper et al., 1995; Branchi et al., 2006). Given that, BDNF is a potential factor throughout social relationships impact cognition during aging (Salinas et al., 2017). The association between BDNF and other social support elements was not observed and the relationship with family support remains under investigation.

We aimed to determine the relationship between family support, cognitive performance and BDNF levels. We assessed family and community support in a population-based cohort of Brazilian older adults without dementia medical diagnosis to test the hypothesis that those who report more significant family support (e.g., structural and functional) and community support (e.g., received or provided social assistance to others) have better cognitive performance and higher BDNF levels.

## Materials and Methods

### Study Design, Setting and Participants

The present study used Brazilian data from the Health, Wellbeing and Aging Study (SABE), a multicenter study started in 2000 by the Pan American Health Organization to determine older adults’ health and living conditions in seven urban centers of Latin America and the Caribbean (Albala et al., 2005). In Brazil, the SABE study was conducted in Sao Paulo, the most populous city in the country, with the highest absolute number of older people, featured by greater diversity due to immigration and internal migration (Lebrao et al., 2019).

In Sao Paulo, SABE was transformed into a multi-cohort longitudinal study to identify changes in the aging process among different generations. Since 2000, 3,257 Brazilian individuals aged 60 years or older, residing in the urban area of the city of Sao Paulo, distributed in four cohorts (A, B, C, and D), were selected through a probabilistic sampling of households registered on census sectors. Visited households were randomly selected through conglomerate sampling. Institutionalized older adults were not included in the SABE study. The first cohort of participants (cohort A in 2000, *n* = 2,143) was re-evaluated every five years, when new cohorts of individuals aged 60 to 64 were added (cohort B in 2006, cohort C in 2010, cohort D in 2015). All data was obtained through an in-person survey at the participant’s house, conducted by trained interviewers and healthcare personnel. Data was composed of health and living conditions assessment, anthropometric and functional evaluation, caloric expenditure, and blood sampling for biochemical and genetic variables. In 2015, new biochemical and immunological variables were incorporated, including BDNF. Additional details about sampling and recruitment are available elsewhere (Lebrao et al., 2019).

The present study was based on a secondary cross-sectional analysis of cohort D data (*n* = 386). Only completed data of family functionality, social support, cognitive performance, and BDNF concentration were considered. Data from participants with dementia diagnosis (family report of medical diagnosis or treatment to dementia, including Parkinson’s disease) was excluded.

The study was approved by the Human Research Ethics Committee of the University of Sao Paulo Faculty of Public Health (#COEP/23/10), São Paulo, Brazil. All participants provided informed consent and identifying information of participants has been anonymized.

### Measures

#### Family Support

The number of people who lived with the participants, whether they live alone and stay alone most of the time, was used to assess the structural component of family support (e.g., size and frequency of interactions). To evaluate the functional dimension of family support, participants were asked to answer five questions related to family function through the Family APGAR questionnaire. APGAR assesses functional components of Adaptability, Partnership, Growth, Affection, and Resolve through five questions designed to reflect the participant’s view of the functional status of his/her family. For each question, the participant should rate from 0 (never) to 4 (always) he/she is satisfied with his/her family partnership, empathy, support, affection and interaction. The total score, which ranges from 0 to 20 points, is the sum of points for each question. Higher scores mean a highly functional family (Smilkstein et al., 1982). Family Apgar assesses participants’ emotional component of functional family support (e.g., loving and empathy relationship).

#### Community Support

Community support was assessed by asking participants whether they received social assistance from community programs such as social service, elderly well-being centers, colleges or universities, a healthy system, and religious entities. They were also asked to indicate whether they provide community support by serving any of those programs in the last 12 months. Data was recorded as a dichotomic variable (yes or no) for each question about receiving and serving in community programs.

### Brain-Derived Neurotrophic Factor Concentration

Blood samples were obtained at fasting morning within 15 days before the survey, at participants’ house through forearm venipuncture using vacuum tubes, free of anticoagulants. Within 1 h after sampling, blood samples were centrifuged at 4000 × *g* for 10 min, and serum was frozen at –80°C until further analysis. Serum BDNF concentration was determined using the commercial Chemikine BDNF ELISA kit (MerckMillipore, Darmstadt/Germany), following the manufacturer’s instructions. Briefly, microtiter plates (96-well flat-bottom) were coated for 24 h at 4°C with the samples diluted 1:100 in sample diluent and standard curve ranging from 7.8 to 500 ng/ml of BNDF (Corrêa et al., 2015). Plates were then washed four times with wash buffer followed by the addition of biotinylated mouse anti-human BNDF monoclonal antibody (diluted 1:1000 in sample diluent), which was incubated for 3 h at room temperature (Corrêa et al., 2015). After washing, a second incubation with streptavidin-horseradish peroxidase conjugate solution (diluted 1:1000) for 1 h at room temperature was carried out (Corrêa et al., 2015). After adding substrate and stop solution, the amount of BDNF was determined (absorbance set at 450 nm) (Corrêa et al., 2015). Samples were analyzed in duplicate, and the intra and inter-assay coefficients of variation were 5.2% and 10.4%, respectively.

### Outcome Measures

#### Cognitive Performance

Participants was evaluated using the Mini-Mental State Examination (MMSE) for global cognition, the semantic verbal fluency (number of animals per minute) and Digit Span Backward for working memory (five digits to be repeated in the reverse order, scoring five points in maximum) (Caramelli et al., 2007; Brucki and Nitrini, 2010).

### Potential Confounders Variables

Possible confounding variables of the association between family function, social support, BDNF and cognitive performance included: (1) sociodemographic characteristics (age, sex, race, education level, and marital status), chronic diseases (self-report of hypertension, diabetes, coronary artery disease, heart failure, pulmonary disease, and stroke), use of medication, smoking (current or previous), alcohol abuse (Michigan Alcohol Screening Test > 2), physical activity (self-report of regular physical activity in the last three months) and depression symptoms (Geriatric Depression Scale with 30 questions – GDS-30) (Yesavage et al., 1982). GDS-30 is a self-report instrument used to identify symptoms of depression in older adults. Participants were asked to answer “yes” or “no” for how they felt regarding enjoyment, interest, and social interactions over the past week. Positive or negative answers that indicate the presence of depression scored 1 point. Geriatric Depression Scale scores range from 0 to 30 points (Yesavage et al., 1982).

### Statistical Analysis

We used mean, standard error (SE), and relative frequencies to describe sample characteristics, chronic diseases, use of medication, smoking, alcohol abuse, physical activity, family structure and community support. The association between cognitive performance and family and social support was tested using the Wald test (adjusted for sample weighted). To investigate the association between cognitive performance (e.g., MMSE, Verbal fluency, and Digit Span) and Family APGAR, we first examined the Pearson correlation coefficients between these variables followed by linear regression models adjusted for the potential confounders (as listed previously). Associations with a p-value of 0.2 or less in the univariate analysis were selected for the multiple regression analysis, in which forward selection was used (Bursac et al., 2008). The Stata 10^®^program (StataCorp, College Station, Tx) was used for all data analysis. The level of statistical significance was set at 0.05 and 95% CI.

## Results

### Participant Characteristics

Participants were predominantly female, between 60 and 64 years old, and with a medium education level. Hypertension and diabetes were the most chronic diseases reported, and almost 20% had a smoking history. Most older adults lived with their partner and children, who used to help them with housework, outside activities, and self-care when necessary. However, almost 20% of them stayed alone most of the time during the day. The majority did not use community support, but more than a quarter served their community (Table 1).

**TABLE 1:** Demographic characteristics, health evaluation and cognitive performance of the participants.

	Sample n	Mean (SE) or %	95% CI Min. – Max.
**Demographic characteristics**			
Age (years)	386	63.2 ± 0.98	62.96114 – 63.35825
Education status (years)	374	9.8 ± 0.28	9.301107 – 10.4566
Sex (% female)	386	54.4	–
Race (%)	386		
White		50.8	–
Brown		38.2	–
Black		8.2	–
Others		2.8	–
Marital status (%)	386		–
Divorced		15.0	–
Widowed		11.6	–
Married		66.2	–
Single		7.2	–
**Health evaluation**			
Hypertension (% yes)	386	57.2	–
Diabetes (% yes)	386	25.5	–
Cardiac disease (% yes)	386	17.7	–
Chronic Pulmonary disease (% yes)	386	8.7	–
Stroke (% yes)	386	5.8	–
Medication use (% yes)	386	9.0	–
Current Smoking (% yes)	386	19.7	–
Alcohol abuse (% yes)	386	5.2	–
Physical activity (% yes)	386	33.8	–
Geriatric Depression scale	374	10.2 ± 0.14	9.952732 – 10.52298
**Cognitive performance and BNDF**			
Mini-Mental State Exam	377	25.0 ± 0.29	24.41897 – 25.60125
Verbal fluency	382	14.4 ± 0.31	13.74534 – 15.00092
Digit span	385	4.0 ± 0.13	3.753055 – 4.279692
BDNF (ng/ml)	309	29.1 ± 11.1	27.74795 30.45865
**Structural and Functional Family Support**			–
Number of people living together	386	2.9 ± 1.6	2.746828 3.128288
Live alone (% yes)	386	12.1	–
Stay alone most of the time during the day (% yes)	386	22.1	–
Family Apgar	382	16.7 ± 0.27	16.2 17.3
**Community Support**	385		–
Receive community support (% yes)		4.8	–
Serve the community (% yes)		27.3	–

### Association Between Social Support, Brain-Derived Neurotrophic Factor, and Cognitive Performance

As previously described, social support was assessed through community support (receiving and offering) and family support (structural and functional). Regarding community support, the participants who offered support in their community showed higher verbal fluency, digit span, and MMSE scores than those who did not serve. No difference was observed between those who received or not received support from the community (Table 2). Concerning the Structural Family Support, the older adults who live alone or stay alone most of the time showed similar BDNF levels and scores on cognitive tests compared to those who did not (Table 2). Similarly, family size (another component of structural family support) was not correlated with BDNF, verbal fluency, and digit span (Table 3). However, for family function component, the higher the Family Apgar, the higher the MMSE, Verbal fluency, and digit span scores (Table 3). Moreover, the multivariate analysis showed that MMSE, verbal fluency, and digit span scores’ variability were significantly explained by the family Apgar, even controlling for age, education, sex, and depression symptoms (Table 4).

**TABLE 2:** Association between family structure, community support, BDNF and cognitive performance (*n* = 386).

	Structural family support						Community support					
Variables	Lives alone mean (± SD)			Stays alone mean (± SD)			Receives support mean (± SD)			Offers support mean (± SD)		
	yes	no	*p*	Yes	no	*p*	yes	No	*p*	Yes	No	*p*
BDNF ng/ml	27.8 (10.1)	29.3 (11.4)	0.419	30.7 (11.5)	28.7 (11.2)	0.164	31.2 (13.3)	29.0 (11.2)	0.547	29.8 (10.1)	28.8 (11.8)	0.490
Verbal fluency	13.9 (4.6)	14.4 (4.1)	0.513	13.8 (4.3)	14.5 (4.1)	0.260	11.7 (4.9)	14.4 (4.1)	0.180	15.4 (4.3)	13.9 (3.9)	0.006
Digit Span	4.3 (1.6)	3.9 (1.6)	0.07	4.2 (1.5)	3.9 (1.6)	0.272	4.0 (1.4)	3.9 (1.6)	0.983	4.4 (1.2)	3.8 (1.7)	<0.001
MMSE	25.7 (3.0)	24.9 (3.9)	0.138	25.4 (3.3)	24.8 (4.0)	0.213	24.4 (3.0)	25.0 (3.9)	0.562	26.6 (3.0)	24.4 (4.0)	<0.001

*MMSE, Mini-Mental State Exam; BDNF, Brain Derived Neurotrophic Factor – *n* = 309.*

**TABLE 3:** Pearson’s correlation coefficients between Family APGAR, BDNF, cognitive performance and potential covariates.

	Correlation’s coefficient								
	Verbal fluency	Digit Span	MMSE	Age	Education	GDS	BDNF	Family Apgar	Family size
	1	2	3	4	5	6	7	8	9
**1**	–								
**2**	0.212**	–							
**3**	0.298**	0.495**	–						
**4**	–0.055	0.016	–0.012	–					
**5**	0.187**	0.238**	0.323**	–0.048	–				
**6**	0.103*	0.048	0.173	–0.043	0.108*	–			
**7**	0.032	–0.058	0. 024	0.065	–0.114*	0.035	–		
**8**	0.236**	0.183**	0.235**	–0.009	0.085	0.247**	0.010	–	
**9**	–0.048	–0.061	–0.134**	0.051	–0.095	–0.047	–0.025	0.026	–

*p < 0.05 and **p < 0.001. Family size = number of relatives living together with the participant. MMSE = Mini-Mental State Exam; Geriatric Depression Scale; BDNF^*a*^ = Brain Derived Neurotrophic Factor – n = 309.

**TABLE 4:** Multivariate regression coefficients between Family APGAR, BDNF and cognitive performance, adjusted for covariates.

Variables	MMSE *n*^1^ = 287 *n*^2^ = 362					Verbal fluency *n*^1^ = 292 *n*^2^^=^ 370					Digit Span *n*^1^ = 298 *n*^2^^=^ 370				
	B	*p*	R^2^	F	*P*	B	*p*	R^2^	F	*P*	B	*p*	R^2^	F	*p*
**Model 1**			0.98	1480.5	<0.001			0.93	342.6	<0.001			0.89	384.9	<0.001
Age	0.249	< 0.001				0.102	0.001				0.034	0.001			
Education	0.365	< 0.001				0.267	0.012				0.102	0.006			
Sex	0.106	0.742				0.394	0.444				0.248	0.218			
GDS	0.192	0.068				0.069	0.557				0.023	0.696			
Family APGAR	0.189	< 0.001				0.214	< 0.001				0.053	0.010			
BNDF^*a*^	0.023	0.160				0.026	0.185				0.003	0.738			

**Model 2**			0.98	3669.5	< 0.001			0.88	767.6	< 0.001			0.88	612.8	< 0.001
Age	0.294	< 0.001				0.132	< 0.001				0.034	< 0.001			
Education	0.374	< 0.001				0.229	0.012				0.110	0.001			
Family APGAR	0.177	< 0.001				0.221	0.001				0.048	0.004			

*Model 1 adjusted for all covariates; n^1^ = number of participants in model 1; Model 2 adjusted only for covariates with *p* < 0.2; n^2^ = number of participants in model 2; GDS = Geriatric Depression Scale; BDNF^*a*^ = Brain Derived Neurotrophic Factor.*

## Discussion

Using data from a population-based cohort, we explored the associations between family and community support and cognitive performance. A key finding was that functional family support, but not structural, was associated with better global cognition, verbal fluency and working memory, even controlling for potential cofounders. A second key finding was that providing support to the community, rather than receiving support from others, was associated with better cognitive performance. Brain-derived neurotrophic factor concentration was not associated with community support, family function, or cognitive performance. These findings suggest that emotional components of functional family and community support (e.g., loving and empathic relationship) may be more significant to cognitive health than size and frequency of social interactions.

Postulated mechanisms through which social relationships might impact cognitive aging include qualitative aspects of one’s social relationships (Southwick et al., 2005). In that regard, our finding demonstrated that the higher the participant’s satisfaction with their family partnership, empathy, support, affection and interaction, the better their cognitive performance. Contradicting other studies with the U.S. and Chinese population (Sims et al., 2014; Ge et al., 2017; Li et al., 2019), our findings that high emotional support was associated with better cognitive performance is consistent with much evidence reporting protective effects of functional support on cognition (Seeman et al., 2001; Windsor et al., 2014; Kelly et al., 2017; Ying et al., 2020; Costa-Cordella et al., 2021). Social interactions are cognitively and emotionally processed by the brain, socially supportive interactions may contribute to better cognitive aging through direct positive stimulatory effects on the brain (Seeman et al., 2001). In line with that, social networks, community engagement are associated with a lower risk of cognitive impairment and dementia (Salinas et al., 2017).

In contrast to previous studies, we did not find an association between cognitive performance and instrumental social support (i.e., receiving assistance/help from others). However, we observed that participants who provided support to their community showed higher performance on global cognition, verbal fluency and working memory performance, suggesting different effects of social support and social activity on specific cognitive domains. Supporting that interpretation, social support was demonstrated to be associated with benefits to episodic memory (Hughes et al., 2008; Kelly et al., 2017), which was not assessed in participants from the SABE study. While social activity (i.e., develop any activity in the community) was most consistently associated with improvements in global cognition and working memory (Plehn et al., 2004; Kelly et al., 2017), that are cognitive domains essential for navigating the complexities of the social world demands (Meyer and Lieberman, 2012). In addition, altruistic behavior seems to have a role in older adults’ cognition, with more altruistic characteristics associated with greater global cognition and verbal fluency, while volunteering was not (Correa et al., 2019). One explanation for the different effects of social support versus social activity may be the impact of social support on stress. Social support has been shown to promote resilience against the negative consequences of stress (Ozbay et al., 2007), whereas simply engaging in social activities or reporting a larger network of family and friends may not translate to the kind of social-emotional support required to obtain such stress-reducing benefits. To promote brain health, our findings allied to previous studies highlight that social support and engagement in social activity may differently impact cognitive domains depending on whether the intervention purpose is to reduce stress or to produce brain stimulation.

Regarding the biological mechanisms explaining how social support and social relationships influence cognition, we did not confirm the hypothesis that higher BDNF levels are associated with higher social support and better cognitive performance. In contrast, Salinas et al. (2017) observed that social isolation trended with lower serum BDNF after controlling for age and sex; meanwhile, having someone available to provide emotional support most or all of the time was associated with higher BDNF and lower dementia risk (Salinas et al., 2017). Although serum BDNF is considered an indirect measure of central nervous system levels (Laske et al., 2007), peripheral pro-BDNF/mBDNF ratio (Cechova et al., 2020), or postmortem BDNF levels may provide different findings. In addition, BDNF changes over time may better capture the protective role mediating social activity and cognitive functioning (Salinas et al., 2017).

Although this study has provided some interesting findings, certain limitations must be considered in interpretating results. First, the associations in this study were based on a cross-sectional design, and reverse causation interpretation is a possibility. Patients with mild cognitive impairment, which is underdiagnosed in the elderly community (Sternberg et al., 2000; Brayne et al., 2007), tend to lose social ties and engagement in community activities. Thus, participants with better cognitive performance were more likely to participate in social activities and provide community support than those with worse cognitive abilities. On the other hand, those participants would also be more likely to receive social support throughout the health system and community entities to handle their cognitive losses, which was not observed in the present study. Furthermore, no measures BDNF gene variants were analyzed. Existing data support a possible effect of BDNF polymorphisms, mainly C270T and Val66Met on the risk of Alzheimer’s disease (AD) (Kunugi et al., 2001; Riemenschneider et al., 2002; Ventriglia et al., 2002). Moreover, no measures of stress were evaluated and should be considered in future studies as both social support and cognition may be influenced by psychological and biological mediators of stress (Juster et al., 2010). Finally, longitudinal data from the SABE study may confirm whether better cognitive performance related to social support at the begging of aging constitutes a protective factor and predictor of successful cognitive aging in later life.

Despite these limitations, our study is unique regarding several relevant aspects. The current findings extend previous research by showing that functional social support (i.e., quality of family relationship and community activity) is a better predictor of global cognition and executive function performance than the structural dimension of social support (i.e., size and frequency of interaction). Moreover, data was based on probabilistic sampling from the most populous cities in Latin America, where the prevalence of dementia is projected to increase dramatically in the next few years (Ferri and Jacob, 2017). Heterogenous education and income backgrounds, allied to cultural characteristics of family ties, whose relationships extend the nuclear members, represent unique social contexts to investigate interaction opportunities and understand its impact on cognition. This study may represent an initial attempt to explore further social and family factors capable of influencing cognitive performance and successful aging.

In summary, our study revealed that, compared to structural social support, function family support and community activity are associated with better global cognition and executive function performance. These findings may inform future studies to investigate whether family relationships and support represent a protective factor for the risk to develop cognitive impairment and dementia in more vulnerable populations.

## Data Availability Statement

The raw data supporting the conclusions of this article will be made available by the authors, without undue reservation.

## Ethics Statement

The study was approved by the Human Research Ethics Committee of the University of Sao Paulo Faculty of Public Health (#COEP/23/10), São Paulo, Brazil. All participants provided written informed consent.

## Author Contributions

JS-T and EB designed and wrote the study protocol, managed the literature searches and analyses, and wrote the final draft of the manuscript. JS and YD are the SABE study PIs, coordinate all study steps from sampling, recruitment, data collection, data management, and data analysis. BF and DS manage literature searches, manage data, and performed BDNF analysis. All authors critically reviewed and approved the final version of the manuscript.

## Conflict of Interest

The authors declare that the research was conducted in the absence of any commercial or financial relationships that could be construed as a potential conflict of interest.

## Publisher’s Note

All claims expressed in this article are solely those of the authors and do not necessarily represent those of their affiliated organizations, or those of the publisher, the editors and the reviewers. Any product that may be evaluated in this article, or claim that may be made by its manufacturer, is not guaranteed or endorsed by the publisher.
